# Chicago Veterinary Society

**Published:** 1900-04

**Authors:** Jos. B. Clancy

**Affiliations:** Secretary


### CHICAGO VETERINARY SOCIETY.

On Monday evening, March 12th, the regular monthly meeting of the Society was called to order by President Hughes, in the hall 912 Masonic Temple. Twenty-seven of the members were present, all of whom aided in bringing about a very entertaining and enjoyable evening.

The report of the special committee instructed to wait upon the Telephone Company and learn why veterinarians are not granted the same privilege that physicians are in the free use of drug-store telephones, was presented by Dr. E. L. Quitman. He said the company allows physicians to use public telephones, by request of drug-store keepers, in payment for the space occupied in their stores.

A paper on "Post-mortem Inspection of Cattle" was presented by Dr. James G. Fish. All present participated in a prolonged and very interesting discussion.

Dr. A. C. Worms denounced the State Veterinary Law as a piece of worthless legislation gotten up for the benefit of a few "grafters," which provoked an extended discussion.

The regular monthly meeting of the Society was called to order by President Hughes on Monday evening, April 9th, in the hall 912 Masonic Temple. Twenty-five members of the Society were present, and a very interesting evening was spent. Following the regular order of business, Dr. L. A. Merillat presented a very valuable contribution on "Belladonna," leaving very little open for discussion. All were pleased and highly interested in the paper, which was discussed by Drs. Robertson, A. H. Baker, and Dr. Hughes. Drs. Merillat and Robertson entered upon quite a lengthy debate on numerous subjects, from belladonna to electricity and palmistry.

President Hughes introduced Professor Mauerman, of Northwestern University, who spoke and showed slides of actinomycosis. During the discussion of Dr. Fish's paper at the March meeting the percentage of tuberculous udders found upon post-mortem was brought up; many of the inspectors have failed to differentiate between tuberculosis and actinomycosis on clinical examination; one of the udders in question was submitted to Dr. Mauerman and found to be actinomycosis on microscopical examination. After a highly interesting discussion it was resolved to adjourn.

Jos. B. Clancy,
Secretary.

